# shRNA-Mediated *XRCC2* Gene Knockdown Efficiently Sensitizes Colon Tumor Cells to X-ray Irradiation *in Vitro* and *in Vivo*

**DOI:** 10.3390/ijms15022157

**Published:** 2014-01-29

**Authors:** Qin Wang, Yan Wang, Liqing Du, Chang Xu, Yuanming Sun, Bing Yang, Zhijuan Sun, Yue Fu, Lu Cai, Saijun Fan, Feiyue Fan, Qiang Liu

**Affiliations:** 1 Tianjin Key Lab of Molecular Nuclear Medicine, Institute of Radiation Medicine of Chinese, Academy of Medical Science and Peking Union Medical College, Tianjin 300192, China; E-Mails: wangqin@irm-cams.ac.cn (Q.W.); wangyan@irm-cams.ac.cn (Y.W.); dlq@irm-cams.ac.cn (L.D.); xuchang@irm-cams.ac.cn (C.X.); sunyuanming@irm-cams.ac.cn (Y.S.); sunzj@irm-cams.ac.cn (Z.S.); wddr0710@gmail.com (Y.F.); fansaijun@irm-cams.ac.cn (S.F.); 2 Department of Cell Biology, Tianjin Medical University, Tianjin 300070, China; E-Mail: yangbingtj@aol.com; 3 Department of Pediatrics of University of Louisville, Louisville, KY 40202, USA; E-Mail: lu.cai@louisville.edu

**Keywords:** XRCC2, RNA interference, colon cancer, ionizing radiation, radiosensitivity

## Abstract

Colon cancer is one of the most common tumors of the digestive tract. Resistance to ionizing radiation (IR) decreased therapeutic efficiency in these patients’ radiotherapy. XRCC2 is the key protein of DNA homologous recombination repair, and its high expression is associated with enhanced resistance to DNA damage induced by IR. Here, we investigated the effect of *XRCC2* silencing on colon tumor cells’ growth and sensitivity to X-radiation *in vitro* and *in vivo*. Colon tumor cells (T84 cell line) were cultivated *in vitro* and tumors originated from the cell line were propagated as xenografts in nude mice. The suppression of *XRCC2* expression was achieved by using vector-based short hairpin RNA (shRNA) in T84 cells. We found that the knockdown of *XRCC2* expression effectively decreased T84 cellular proliferation and colony formation, and led to cell apoptosis and cell cycle arrested in G2/M phase induced by X-radiation *in vitro*. In addition, tumor xenograft studies suggested that *XRCC2* silencing inhibited tumorigenicity after radiation treatment *in vivo*. Our data suggest that the suppression of *XRCC2* expression rendered colon tumor cells more sensitive to radiation therapy *in vitro* and *in vivo*, implying *XRCC2* as a promising therapeutic target for the treatment of radioresistant human colon cancer.

## 1. Introduction

Colorectal cancer, including colon and rectal cancer, is one of the most common tumors of the digestive tract. It is the third most commonly diagnosed cancer in males and the second in females, with over 1.2 million new cancer cases and 608,700 deaths estimated to have occurred in 2008 [1,2]. The best therapy for colon cancer is through surgical excision after early diagnosis, which can be combined with radiotherapy, chemotherapy, and immunotherapy. Radiotherapy before surgery makes tumor volume shrink and improves the removal rate. Radiotherapy after surgery kills remaining tumor cells. Radiotherapy alone is suitable for colon cancer patients in advanced stage, which has the effect of stopping bleeding and pain. Collectively, radiation plays an important role in colon cancer’s therapy. However, colon cancer is not sensitive to radiotherapy, prompting to the search of novel strategies to enhance radiosensitivity of colon cancer for reducing cancer recurrence after surgery and improving survival of patients.

The inactivation of tumor suppressor genes, activation of oncogenes or various repair genetic defects in DNA replication is closely associated with colorectal cancer incidence [3]. The double-strand break (DSB) is the main DNA lesion induced by ionizing radiation (IR). Unrepaired DSB or misrepaired DSB may lead to cell death or mutation or carcinogenesis. The two methods of repairing DSB include homologous recombination (HR) and non-homologous end-joining (NHEJ). HR, error-free repair pathway, is an evolutionarily conserved mechanism for the repair DSB without inducing base substitutions, deletions, or insertions. In mammalian cells, repair of DSB via HR is dependent on the RecA/Rad51 family.

X-ray repair cross complementing gene family (*XRCC1*–*XRCC11*) plays an important role in repairing DNA damage induced by IR. *XRCC2* gene, a member of the family, first separated from hamster cells line Irsl, is closely associated with HR. *XRCC2* locates on human chromosome 7q36.1, cDNA 1,580 bp, encodes 280 amino acid, which is a key component of HR repair proteins RecA/Rad51family [4]. *XRCC2* recruits Rad51 to the broken DNA ends. Then, the complex, formed by *XRCC2*, *Rad51B*, *Rad51C*, and *Rad51D*, ligases with single-stranded DNA and DSB, which promotes *Rad51* to identify location of DSB and assembly *Rad51*-DNA filaments. It helps to maintain genomic stability [5] and resist various cytotoxic factors (such as IR) damage to DNA [6]. A report found that *XRCC2* gene deletion caused the formation defects of the core protein RAD51, which resulted in lack of effective repair of DNA damage and increased the spontaneous chromosomal aberrations and chromosomal abnormalities separation [7]. Therefore, *XRCC2* abnormal expression can lead to genomic instability and promote tumor development and progression.

A recent study showed that XRCC2-deficient cells were defective in HR repair function in response to DSB as compared with the parent cell lines [4]. Consequently, the sensitivity to IR is significantly increased in XRCC2-deficient cells than in normal cells [8]. An investigation suggested that the abnormal upregulation of *XRCC2* gene expression rendered lung cancer cells’ resistance to DNA damage induced by radiation, which resulted in tumors’ resistance to radiotherapy [9]. Thus, we propose that the inhibiting of *XRCC2* expression of tumor cells may enhance their radiosensitivity.

In recent years, studies on XRCC2 focuses on HR repair mechanisms and the relationship between *XRCC2* gene polymorphism and tumor susceptibility [10–14], such as the relationship between XRCC2 R188H polymorphism and breast cancer, thyroid cancer, ovarian cancer, *etc.* Reports on XRCC2 associated with colorectal cancer only deal with XRCC2 polymorphism [15–19]. However, there are no studies on *XRCC2* expression in colon cancer and its association with sensitivity to IR. Until now, it is not yet known whether the level of *XRCC2* expression can affect the sensitivity of radiotherapy for colon cancer and whether *XRCC2* can predict the efficacy of colon cancer radiotherapy. Thus, we adopted shRNA-mediated *XRCC2* silencing strategy to investigate the effect of *XRCC2* silencing on cell growth and sensitization to X-radiation in colon cancer *in vitro* and *in vivo*.

## 2. Results

### 2.1. Expression of *XRCC2* in Various Tumor Cell Lines and Normal Cell Line

**Figure 1.**
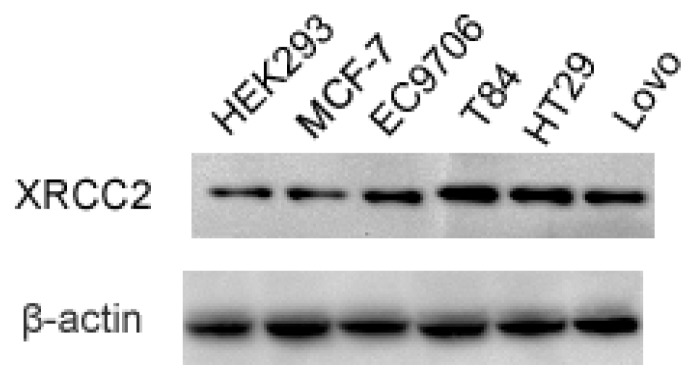
XRCC2 protein was expressed highly in T84 colon cancer cell line. Total protein of various tumor cell lines (colon cancer cell lines T84, HT29 and Lovo, breast cancer cell line MCF-7, esophageal cancer cell line EC9706) and normal HEK293 cell line were extracted, and the *XRCC2* expression was examined by Western blot.

To clarify whether *XRCC2* expression is abnormal in human colon cancer, we analyzed *XRCC2* expression in various tumor cell lines (colon cancer cell lines T84, HT29 and Lovo, breast cancer cell line MCF-7, esophageal cancer cell line EC9706) and normal HEK293 cell line. As shown in Figure 1, overexpression of *XRCC2* appeared in tumor cell lines compared with HEK293. Given the elevated level of XRCC2 protein in T84 colon cancer cell line, we selected T84 in subsequent experiment.

### 2.2. Knockdown of *XRCC2* Using Vector-Based shRNA in T84 Cells

**Figure 2.**
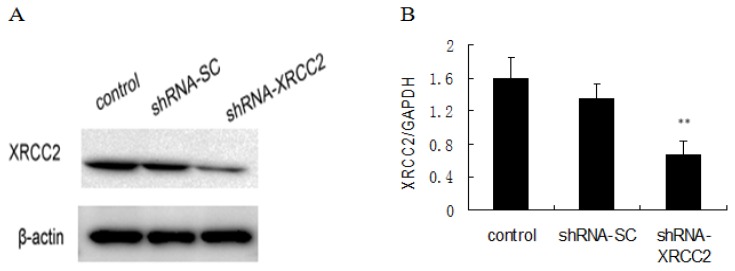
XRCC2 expression was suppressed by vector-based shRNA in T84 cells. (**A**) XRCC2 protein expression; and (**B**) *XRCC2* mRNA expression. Cells were transfected with either shRNA-XRCC2 or shRNA-SC. Total protein and mRNA levels of *XRCC2* were determined at 24 h after transfection by Western blot and quantitative real-time polymerase chain reaction (PCR) analyses, respectively. The values are presented as the mean ± SD (*n* = 6). ** *p* < 0.01 compared with the control group.

RNAi technology was used to knockdown *XRCC2* expression in T84 cells. The vector-based shRNA plasmid (shRNA-XRCC2) were transfected into T84 cells. The scrambled cells were treated with control shRNA plasmid-A as scramble shRNA (shRNA-SC). The efficiency of transfection was evaluated by the levels of *XRCC2* mRNA and protein expression. *XRCC2* expression was lower in shRNA-XRCC2 cells than in shRNA-SC cells (Figure 2), indicating that *XRCC2* expression was effectively suppressed by shRNA-XRCC2.

### 2.3. Effect of *XRCC2* Knockdown on Cell Growth of T84 Cells

**Figure 3.**
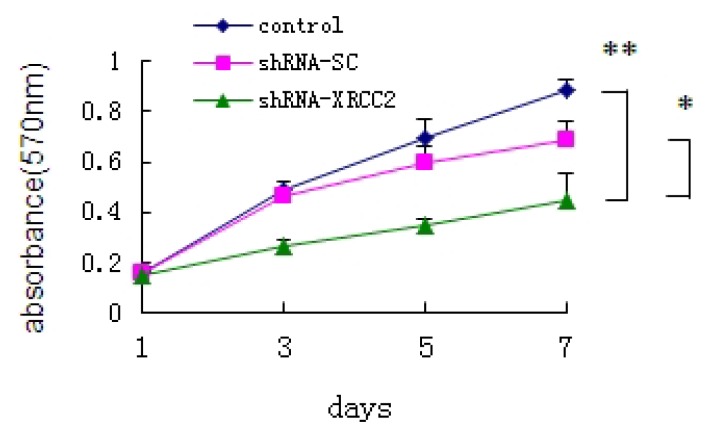
Knockdown of *XRCC2* by shRNA inhibited cell growth of T84 cells. Cells were transfected with either shRNA-XRCC2 or shRNA-SC. The effect of *XRCC2* suppression on cell growth in T84 cell line was examined by MTT assay. The values are presented as the mean ± SD (*n* = 12). * *p* < 0.05, ** *p* < 0.01 compared with the control group.

To investigate the role of *XRCC2* on the growth of T84 cells, we analyzed the growth curve of cells *in vitro* by MTT assay. Cells of control group and shRNA-SC group grew steadily by linear growth. An obvious decrease in the cell proliferation rate was found on the third day in the shRNA-XRCC2 cells compared with shRNA-SC transfectant and control cells (Figure 3). Results revealed that shRNA-XRCC2 resulted in more efficient inhibition of cell growth than shRNA-SC.

### 2.4. Effect of *XRCC2* Knockdown on the Response of T84 Cells to X-Radiation

**Figure 4.**
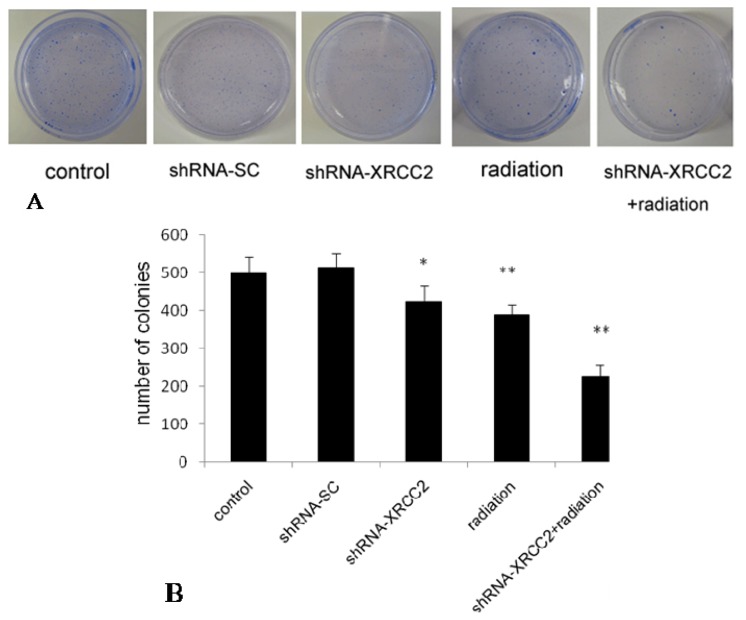
Knockdown of *XRCC2* by shRNA enhanced radiosensitivity to X-radiation of T84 cells. (**A**) Photographs of colonies; and (**B**) the number of colonies. Cells were treated with either shRNA-XRCC2, shRNA-SC, radiation or shRNA-XRCC2 combined radiation. Cells of control group, shRNA-SC group and shRNA-XRCC2 group were not exposed to radiation. Cells of radiation group were treated with 8 Gy X-ray radiation after T84 cells attached. shRNA-XRCC2 combined radiation group received 8 Gy radiation at 12 h after shRNA-XRCC2 transfection. The effect of *XRCC2* suppression on radiosensitivity of T84 cell line was examined by colony formation assay. The values are presented as the mean ± SD (*n* = 6). * *p* < 0.05, ** *p* < 0.01 compared with the control group.

To determine the effect of *XRCC2* knockdown on radiosensitivity to X-radiation of T84 cells, we examined their ability to form colonies *in vitro* (Figure 4). The number of colonies formed by shRNA-XRCC2 cells was significantly decreased compared with that of control cells. However, there was no significant change in shRNA-SC cells compared with control cells. Compared with shRNA-XRCC2 group and radiation group, the number of colonies in shRNA-XRCC2 combined radiation group was decreased significantly. The results suggested that shRNA-XRCC2 cells were more sensitive to radiation than shRNA-SC and control cells.

### 2.5. Effect of *XRCC2* Knockdown on Cell Cycle Distribution Induced by Radiation

**Figure 5.**
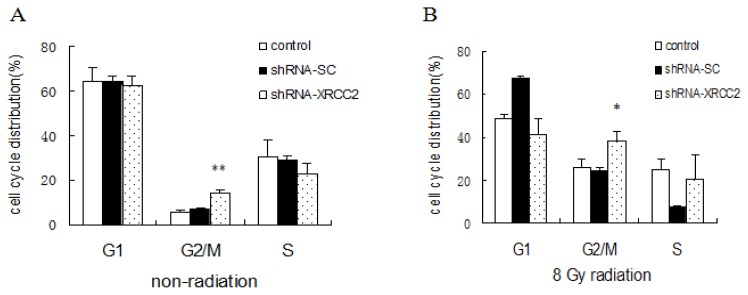
Knockdown of *XRCC2* arrested cells in the G2/M phase. (**A**) Cell cycle distribution without exposure to radiation; and (**B**) cell cycle distribution after exposure to 8 Gy radiation. Cells were transfected with either shRNA-XRCC2 or shRNA-SC. Cells were irradiated with 8 Gy at 12 h after transfection. The effect of *XRCC2* suppression on cell cycle distribution in T84 cell line was examined by flow cytometric analysis. The values are presented as the mean ± SD (*n* = 5). * *p* < 0.05, ** *p* < 0.01 compared with the control group.

We analyzed the relationship between the increased sensitivity of shRNA-XRCC2 cells to IR with the cell cycle distribution. The proportion of shRNA-XRCC2 cells in the G2/M phase was higher than that of shRNA-SC cells and control cells without radiation treatment. When exposed to 8 Gy radiation, the shRNA-XRCC2 cell population in the G2/M phase was significantly increased, and those in the G1 phase was decreased compared with control cells (Figure 5). The results suggested that the knockdown of *XRCC2* enabled XRCC2 tumor cells to escape from G1 phase and to arrest in the G2/M phase.

### 2.6. Effect of *XRCC2* Knockdown on Cell Apoptosis Induced by Radiation

**Figure 6.**
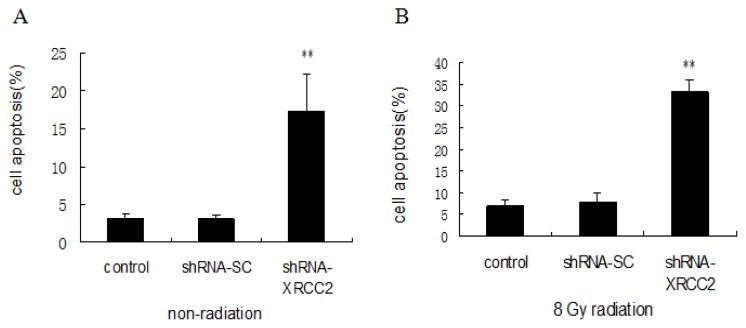
Knockdown of *XRCC2* enhanced cell apoptosis after radiation treatment. (**A**) Cell apoptosis without exposure to radiation; and (**B**) cell apoptosis after exposure to 8 Gy radiation. Cells were transfected with either shRNA-XRCC2 or shRNA-SC. Cells were irradiated with 8 Gy at 12 h after transfection. The effect of *XRCC2* suppression on cell apoptosis in T84 cell line was examined by flow cytometric analysis. The values are presented as the mean ± SD (*n* = 5). ** *p* < 0.01 compared with the control group.

To investigate whether the increased sensitivity of cells transfected with shRNA-XRCC2 after exposure to radiation is related to apoptosis, we examined cell apoptosis by flow cytometry. The number of apoptotic cells of shRNA-XRCC2 group was higher than that of control and shRNA-SC cells without radiation treatment. When irradiated with 8 Gy radiation, the number of shRNA-XRCC2 apoptotic cells was increased compared with that of control and shRNA-SC cells, which showed that shRNA-XRCC2 cells were more sensitive to apoptosis induced by radiation (Figure 6). These findings suggested that the knockdown of *XRCC2* expression mediated radiosensitization of T84 cells, and was associated with enhanced apoptosis.

### 2.7. Effect of *XRCC2* Knockdown on Tumorigenicity in Nude Mice

**Figure 7.**
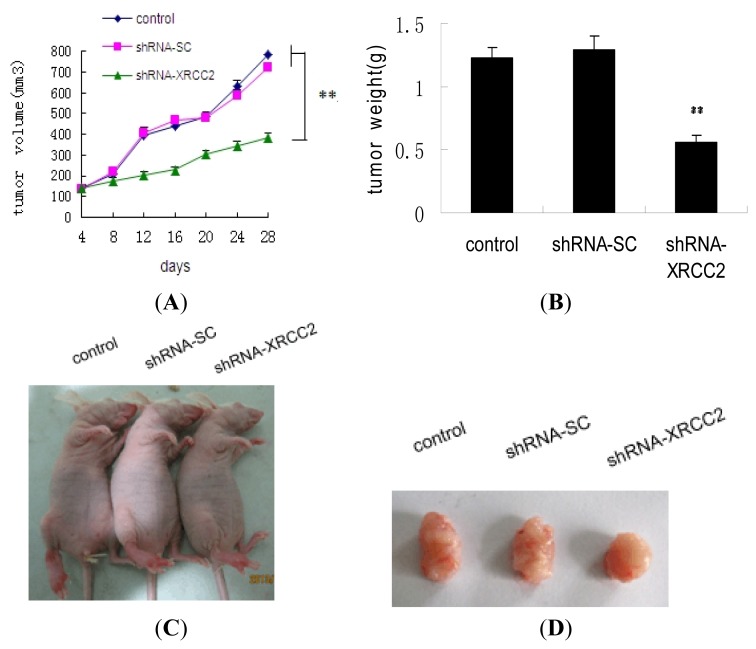
Knockdown of *XRCC2* decreased tumor growth in nude mice. (**A**) Tumor growth curve; (**B**) Tumor weight after mice were sacrificed; (**C**) Ectopic T84 xenografts in nude mice; and (**D**) Photograph of the dissected tumors. The effect of shRNA-XRCC2 on tumorigenicity was examined in nude mice. Mice were treated with either shRNA-XRCC2 or shRNA-SC. Radiation treatment (2 Gy) was given once every other day when tumor volume reached 100 mm^3^ in a total of 10 days. The tumor volume and tumor weight was examined for 28 days after exposure to radiation. The values are presented as the mean ± SD (*n* = 10). ** *p* < 0.01 compared with the control group.

To determine the effect of shRNA-XRCC2 on tumor growth *in vivo*, a xenograft model was performed in nude mice. As shown in Figure 7A, tumors of control group and shRNA-SC group grew abruptly, whereas those of shRNA-XRCC2 group grew steadily. Tumor growth was retarded in shRNA-XRCC2 group compared with control group. At day 28 after exposure to radiation, the tumor volume of shRNA-XRCC2 group was decreased significantly compared with that of control group. In addition, the tumor weight was also lighter in shRNA-XRCC2 group than in control group after mice were sacrificed (Figure 7B). As shown in Figure 7C,D, the tumors of shRNA-XRCC2 transfected cells were obviously smaller than that of shRNA-SC and control cells. The results suggested that the knockdown of *XRCC2* rendered colon tumor cells more sensitive to reradiation.

### 2.8. Effect of *XRCC2* Knockdown on Pathology in Nude Mice

**Figure 8.**
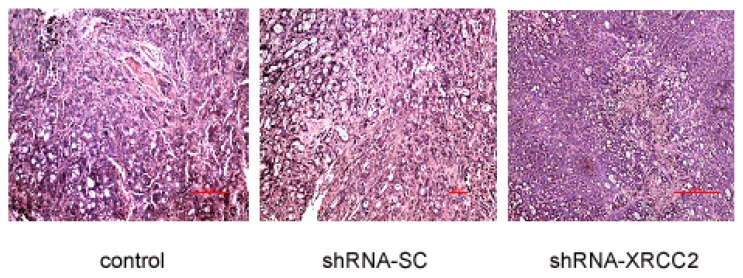
Pathological analysis of knockdown of *XRCC2* expression. The effect of XRCC2 shRNA on pathology was examined in nude mice. Mice were treated with either shRNA-XRCC2 or shRNA-SC. Radiation treatment (2 Gy) was given once every other day when tumor volume reached 100 mm^3^ in a total of 10 days. The tumor pathology was examined on day 28 after exposure to radiation. (Magnification: ×400; Scale bar: 100 μm).

The mice were sacrificed at 28 days after radiation treatment. Tumors were harvested for pathological analysis. The pathological examination revealed detailed effect of *XRCC2* knockdown on tumor growth *in vivo* (Figure 8). Tumor tissue arrangement showed gland-like structure and no obvious capsule was seen in dissected tumors of three groups. Pathological analysis of control group and shRNA-SC group was essentially identical: some inflammatory cells were seen in glandular cavity and infiltrated to peritumor; karyokinetic figure was commonly seen and no obvious necrosis appeared in tumor tissue. However, small areas of necrosis were found in shRNA-XRCC2 group (Table 1). Our data suggested that treatment of shRNA-XRCC2 transfection had better anti-tumor effect than that of control and shRNA-SC.

## 3. Discussion

The resistance to radiation therapy is a critical element of affecting the survival of colon cancer patients, and is attributable to various mechanisms [20,21]. The role of the DNA repair protein XRCC2 on colon cancer radioresistance remains poorly understood. In the study, we adopted vector-based shRNA expression system to investigate the effect of *XRCC2* silencing on sensitization to X-radiation in colon cancer *in vitro* and *in vivo*.

A recent study showed that the incidence of spontaneous breast and intestinal tumorigenesis was higher in XRCC2^+/+^ mice than in XRCC2^+/−^ animals [22], implicating a possible role for XRCC2 in affecting tumor cells’ radiosensitivity. In the present study, we found that the expression of *XRCC2* protein was elevated in colon cancer cells compared with normal cells. As uncontrolled growth is one of important alteration in cancer cell phenotypes, we firstly examined the effect of *XRCC2* knockdown on the growth of T84 colon tumor cells. Our experimental data indicated that the suppression of *XRCC2* expression effectively decreased cellular proliferation *in vitro*. In addition, tumor xenograft studies showed *XRCC2* silencing similarly inhibited tumor growth *in vivo*, suggesting XRCC2 as an important regulator of colon tumor cellular proliferation.

To test this possibility, we studied the effect of *XRCC2* suppression on radiosensitivity of colon cancer cells. We found that shRNA-mediated *XRCC2* suppression rendered T84 tumor cells more sensitive to radiation treatment as evaluated by the colony formation assay. The XRCC2-mediated enhancement of radiosensitivity was associated with an elevation of radiation-induced apoptosis, suggesting a possible role of XRCC2 in the regulation of radiation-induced apoptosis. Our findings were consistent with previous reports that silencing *XRCC2* expression using RNA interference technology similarly improved the radiosensitivity of human brain tumor and lung cancer cells [23]. In addition, our investigation showed that lowering *XRCC2* expression level enhanced the sensitivity of tumors to radiation in nude mice. Collectively, our experimental data suggest that knockdown of *XRCC2* expression enhances colon tumor cells’ sensitivity to radiotherapy *in vitro* and *in vivo*.

The cell cycle phase is one of the most important determinant of radiosensitivity, with cells being most radiosensitive in the G2/M phase, less sensitive in the G1 phase, and least sensitive during the latter part of the S phase. Our results showed that cells transfected with shRNA-XRCC2 were arrested in G2/M phase after exposed to radiation. As cells arrested in the G2/M phase are generally more sensitive to radiation than those in other phases [24], it is plausible that the XRCC2-mediated change of the cell cycle may consequently affect the sensitivity of tumor cells to radiation therapy.

Although several reports have suggested an association between *XRCC2* expression and radiosensitivity [8,9], it is unclear whether suppression of *XRCC2* expression in any tumor cells uniformly facilitates tumor radiotherapy. Similarly, the molecular mechanisms by which *XRCC2* mediates tumor cells’ radiosensitivity remains a subject of future investigation. Given that inhibiting of *XRCC2* expression can enhance radiosensitivity of tumor cells, strategies to knockdown *XRCC2* expression may improve the efficacy of radiation therapy in clinical settings.

In summary, knockdown of *XRCC2* significantly sensitized colon tumor cells to radiation, as assayed in both *in vitro* cell culture and *in vivo* xenograft tumor models. The study suggest that *XRCC2* might be a radiotherapy target in colon cancer, and that *XRCC2* gene-specific therapy may be developed as an assistant method of enhancing the antitumor effect of traditional radiation treatment.

## 4. Experimental Section

### 4.1. Cell Culture

The human colon cancer cell line (T84, HT29 and Lovo) was purchased from the Cell Culture Center of Basic Medicine, Chinese Academy of Medical Sciences, Beijing, China. Breast cancer cell line MCF-7, esophageal cancer cell line EC9706 and normal human embryonic kidney cell line HEK293 were obtained from our laboratory. Cells were cultured in DMEM/F12 (Hyclone, Beijing, China) supplemented with 10% FBS and 100 μg/mL of penicillin/streptomycin, in a humidified 5% CO_2_ incubator at 37 °C.

### 4.2. Ionizing Radiation

Cells and mice were exposed to ionizing radiation (IR) in a X irradiator (Rad source Inc., Suwanee, GA, USA) at a rate of 0.99 Gy/min.

### 4.3. shRNA Plasmid Stable Transfection

T84 cells were stably transfected with either shRNA XRCC2 plasmid (sc-36861-SH, Santa Cruz, Dallas, TX, USA) (shRNA-XRCC2) or control shRNA plasmid-A (sc-108060, Santa Cruz) as scramble shRNA (shRNA-SC), using Lipofectin reagent (Invitrogen, Carlsbad, CA, USA), according to the manufacturer’s instruction. Briefly, 1 μg of each plasmid DNA and 20 μL of Lipofectin 2000 (Invitrogen) were mixed separately with serum-free Optim-MEM medium (Invitrogen) and incubated for 5 min at room temperature. Then they were combined and incubated for 15 min. After 48 h incubation, individual colonies were selected for resistance to 10 μg/mL puromycin for 2 weeks. Signal colonies were isolated and cultured to obtain stably transfected cells line. The effectiveness of transfection was examined by Western blot.

### 4.4. MTT Assay

T84 cells were planted at a density of 1000/well in a 96-well plate in triplicate. After cells adhered, samples were infected with either shRNA-XRCC2 or shRNA-SC and cultured at 37 °C. Each well was added 20 μL MTT (5 μg/mL, Amresco Inc., Solon, OH, USA) on day 1, 3, 5, and 7, and incubated for 4 h. The medium was removed and 150 μL dimethyl sulphoxide (DMSO) was added. The absorbance of each well was measured by the Multi-Mode Microplate Reader (Synergy HT, BioTek, Winooski, VT, USA) at 570 nm.

### 4.5. Colony Formation Assay

A colony formation assay was performed to determine sensitivity of T84 cells to X-radiation. Eight hundred T84 cells were seeded in 55 mm dishes and allowed to attach. Cells were treated with either shRNA-XRCC2, shRNA-SC, radiation, or shRNA-XRCC2 combined radiation. The control group did not receive any treatment. Radiation group were treated with 8 Gy X-ray radiation after T84 cells attached. ShRNA-XRCC2 combined radiation group received 8 Gy radiation at 12 h after shRNA-XRCC2 transfection. Colonies were fixed by methanol and stained with the Giemsa stain at day 14 after incubation. A minimum of 50 viable cells were scored as a colony.

### 4.6. Western Blot Analysis

Cells (1 × 10^6^ cells) were seeded in 100 mm dishes. After cells adhered, samples were infected with either shRNA-XRCC2 or shRNA-SC. Cells were lysed in mammalian protein extraction reagent (M-PER) (Thermo, Waltham, MA, USA). Total protein concentration was determined using a Bicinchoninic acid (BCA) protein assay kit (Beyotime Biotechnology, Shanghai, China). Total protein (30 μg) was loaded onto 10% gradient SDS-PAGE gels and then transferred to polyvinylidene fluoride (PVDF) membranes (Millipore, Billerica, MA, USA). Membranes were blocked and incubated with the following antibodies: The mouse anti-XRCC2 antibody (1:1000 sc-73278) from Santa Cruz Biotechnology Incorporation, rabbit anti-β-actin (1:5000, P30002) and goat anti-mouse IgG conjugated to horseradish peroxidase (M21001) from Abmart Incorporation (Beijing, China). Chemiluminescent signal detection was performed by an electrochemical luminescence (ECL) kit (Boster Biotechnology, Abingdon, UK).

### 4.7. Quantitative Real-Time PCR

Cells (1 × 10^6^ cells) were seeded in 100 mm dishes. After cells adhered, samples were infected with either shRNA-XRCC2 or shRNA-SC. XRCC2 mRNA expression was performed by Quantitative real-time PCR (qPCR). Total RNA was prepared using Trizol reagent (Invitrogen) according to the manufacturer’s instruction. The first strand of cDNA was synthesized using RNA PCR kit (Takara Bio Inc., Dalian, China). qPCR was performed on the CFX96 (BioRad, Hercules, CA, USA) with SYBR Green kit (Takara Bio Inc.) according to the manufacturer’s instruction. The sequences of the primers were as follows: XRCC2 forward 5′-CAATGGAGGAGAAAGTGTGAACT-3′, XRCC2 reverse 5′-CAAAAAGAACCAGGCGATAGTC-3′, GAPDH (set as the reference) forward 5′-CGAATTGGCTACAGCAACAGG-3′, GAPDH reverse 5′-GTACATGACAAGGTGCGGCTC-3′. The primers were obtained from Sangon Biotech (Shanghai, China).

### 4.8. Cell Cycle

Cell cycle distribution was measured by flow cytometric analysis. Cells (1 × 10^5^ cells/well) were seeded in 6-well culture plates. After cells adhered, samples were infected with either shRNA-XRCC2 or shRNA-SC. Radiation treatment was given 8 Gy X-radiation at 12 h after transfection and then further cultured for 24 h. Cells were harvested by trypsinization and fixed in ice-cold 70% ethanol overnight. After washing with PBS, the fixed cells were stained with propidium iodide solution (50 μg/mL propidium iodide, 0.1% Triton X-100, and 0.1% sodium citrate in PBS) for 30 min at 4 °C in the dark. The DNA content was determined by a FACS cytometer (Becton-Dickinson, Franklin Lakes, NJ, USA) using CellQuest program (Becton-Dickinson) and data from ≥10,000 cells were analyzed with ModFit software (Becton-Dickinson).

### 4.9. Cell Apoptosis

Cells apoptosis were determined with FITC annexin V apoptosis detection kit (556570, BD pharmingen, Taipei, Taiwan) using flow cytometric analysis. Cells (1 × 10^5^ cells/well) were seeded in 6-well culture plates. After cells adhered, samples were infected with either shRNA-XRCC2 or shRNA-SC. Radiation treatment was given 8 Gy X-radiation at 12 h after transfection and then further cultured for 24 h. Cells were harvested by trypsinization and incubated in 0.5 mL of binding buffer containing 0.5 μg/mL Annexin-V-FITC and 5 μg/mL propidium iodide for 30 min in the dark. Cells apoptosis was measured by a FACS cytometer (Becton-Dickinson) using the CellQuest program and data from ≥10,000 cells were analyzed with ModFit software.

### 4.10. Tumorigenicity in Nude Mice

Six-week-old male BALB/c nude mice were purchased from Academy Military Medical Sciences (Beijing, China). The experimental protocol was approved by the China Institutional Ethics Review Committee for Animal Experimentation. Cells (1 × 10^7^/mouse) suspended in 0.2 mL DMEM/F12 medium were injected subcutaneously into the right leg of nude mice. The mice were randomly divided into three groups: control, shRNA-SC, and shRNA-XRCC2, five mice in each group. Radiation treatment (2 Gy) was given once every other day when tumor volume reached 100 mm^3^ in a total of 10 days. The tumor volume was measured over the skin from an external caliper using the formula: V (mm^3^) =1/6 π × length (mm) × width^2^ (mm^2^). The nude mice were anaesthetized using chloral hydrate solution (0.3 g/kg) intraperitoneally before radiation was delivered to the tumor area with the rest of the mice shielded. The mice were sacrificed on the twenty eighth day after radiation treatment. Tumors were dissected, weighed, and harvested for pathological analysis.

### 4.11. Pathological Analysis

Dissected tumors were fixed in 4% neutral buffered formalin and embedded in paraffin using standard procedures. Paraffin embedded tumors were cut in 5 μm sections at the maximum cross-section. Sections were stained with haematoxylineosin (H.E.) and analyzed under a microscope (BX51, Olympus, Shinjuku, Japan) at 400-fold magnification.

### 4.12. Statistical Analysis

All experiments were repeated at least two times. Data are presented as mean ± SD. Differences between groups were calculated using the Student’s *t* test. *p-*values < 0.05 were considered as statistically significant.

## 5. Conclusions

In conclusion, knockdown of *XRCC2* expression has radiosensitization effects on colon tumor cells *in vitro* and *in vivo*. These data strongly suggested that *XRCC2* may be further developed as a promising therapeutic target for the treatment of radioresistant human colon cancer.

## Figures and Tables

**Table 1.** Pathological analysis of harvested tumors in nude mice.

	Control	shRNA-SC	shRNA-XRCC2
Tumor margin	+	+	+
Architectural atypia	+	+	+/−
Cellular atypia	+	+	+
Karyokinesis	++	++	+
Necrotic area	+/−	+/−	+
